# Pose-aware deep perceptual similarity for iterative 2D/3D registration of knee joints using contrastive learning

**DOI:** 10.1007/s11548-026-03734-9

**Published:** 2026-06-19

**Authors:** Jinhao Wang, Xia Li, Raphael Surbeck, Saša Ćuković, William R. Taylor

**Affiliations:** 1 Laboratory for Movement Biomechanics, Institute for Biomechanics, Department of Health Sciences and Technology, ETH Zürich, Zürich, Switzerland; 2 Institute for Biomedical Engineering, ETH Zürich, Zürich, Switzerland

**Keywords:** Similarity metric, 2D/3D registration, Joint kinematics, Optimization

## Abstract

**Purpose:**

Accurate 2D/3D registration between fluoroscopic X-ray images and preoperative CT models is essential to get dynamic joint kinematics in computer-assisted orthopedic analysis. Traditional correlation-based similarity metrics in model–image optimization often suffer from limited capture range and sensitivity to noise and occlusion.

**Methods:**

We propose a pose-aware deep perceptual metric using contrastive learning for enlarged capture range and improved robustness. Inspired by LPIPS, our network was specialized and designed for 2D/3D fluoroscopic image registration, where the supervision reflects true 3D transformation discrepancies. The learned metric is integrated into a differentiable 2D/3D registration framework, enabling gradient-based optimization.

**Results:**

The proposed metric enlarges the capture range of 2D/3D registration and smooths the loss landscape, while ensuring the required accuracy for automated registration. Our metric outperforms conventional similarity measures and standard LPIPS, presenting zero-shot ability for unseen low-contrast fluoroscopic conditions.

**Conclusion:**

The proposed metric provides a robust and differentiable metric for fluoroscopic 2D/3D registration, improving accuracy in joint kinematics analysis for both single- and dual-plane fluoroscopy.

## Introduction

Fluoroscopic imaging has become one of the most powerful tools for in vivo assessment of joint kinematics [1–4], offering high temporal resolution and the ability to capture *in vivo* kinematics under functional loading without invasive markers [5]. These advantages make fluoroscopy indispensable in computer-assisted orthopedic analysis and biomechanical research.

A fundamental step in reconstructing accurate 3D kinematic information from fluoroscopy is the rigid 2D/3D registration. which aligns fluoroscopic X-ray images with patient-specific CT-derived bone or implant models. For each subject, the femur and tibia are segmented from preoperative CT and converted into subject-specific 3D models. During motion trials, dynamic fluoroscopic image sequences are acquired. For each fluoroscopic frame, the 3D femur and tibia models are rigidly aligned to the 2D X-ray image, yielding a time-resolved estimate of the 3D pose of each bone. These frame-wise poses are then used to reconstruct in vivo knee joint kinematics. The accuracy of 2D/3D registration directly determines the reliability of reconstructed joint motion, particularly in applications such as knee replacement evaluation, implant tracking, and dynamic musculoskeletal analysis.

Traditional rigid 2D/3D registration frameworks for joints typically follow two major strategies: deep learning-based pose prediction [6–13] and model–image optimization [14–16]. Deep networks can provide an initial poses and significantly reduce the search space [17], but they rarely achieve the submillimeter and sub-degree accuracy required for quantitative kinematic analysis. Consequently, most high-precision pipelines still rely on a subsequent optimization-based refinement stage, where the model–image similarity is iteratively optimized until converged. However, achieving robust refinement in real fluoroscopy is challenging. Model–image optimization depends heavily on the choice of image similarity metrics measured between target X-ray and digitally reconstructed radiography (DRR) from CT segments. Conventional similarity measures [18], such as LNCC (local normalized cross-correlation) [15], GCC (gradient correlation coefficient) [19], MIND [20], and even hybrid metric [21], often lack discriminative power in low-dose, noisy, and artifact-prone fluoroscopic images. As a result, the optimization loss landscape becomes highly non-convex, and the convergence becomes prone to local minima when the initialization deviates little from the true pose [22]. Such issues from realistic scenarios limit the accuracy and capture range required in automating a reliable 2D/3D rigid registration.

Recent advances in learning-based similarity have shown encouraging progress for image registration tasks. Early perceptual metrics such as LPIPS [23] or DISTS [24] leveraged CNNs to extract high-level visual features that correlate with structural similarity, while more recent works explore using intermediate layers of diffusion models [25] to encode richer semantic or geometric representations. However, these metrics are designed to handle 2D–2D image discrepancy instead of the essential 3D pose awareness in 2D/3D registration. Other studies have investigated semantic registration [26, 27], in which modality-invariant features are extracted from multimodal medical images to facilitate anatomical alignment. Some of these methods rely on large pre-trained semantic encoders, which can provide strong general representations and may be compatible with iterative optimization. However, such approaches are not explicitly designed to encode rigid pose discrepancy in fluoroscopy-to-CT 2D/3D registration, and their learned feature distances are not directly supervised to preserve geometry-consistent ordering with respect to 3D pose misalignment. Representation learning-based methods employing contrastive objectives have also demonstrated the ability to learn discriminative embeddings for structural correspondence [28–30]. Gao et al. [31], for instance, proposed a deep similarity module integrated into an end-to-end differentiable registration network using geodesic distance. Nevertheless, their approach is tightly coupled to the their pipeline specified for their problem. The generalization of such methods are hardly to be extended to our problem, and their similarity lacks modularity. Despite their successes, existing perceptual similarity networks are typically trained on natural images and do not reflect the radiographic physics, occlusions, or pose sensitivity inherent to fluoroscopy.

As a result, existing metrics cannot implicitly constructs a convex similarity landscape that generalize across rigid pose variations especially under noisy imaging conditions, thereby limiting their usefulness as objective functions for model–image optimization. These limitations motivate the development of a fast and pose-aware similarity metric whose value increases smoothly with rigid transformation error and maintains a convex, well-behaved landscape even far from the ground-truth pose.

To achieve such similarity, we present a pose-aware deep perceptual similarity metric inspired from contrastive learning [29, 30] and geodesic distance learning [31] for our fluoroscopy-to-CT 2D/3D registration. Our method adapts a differentiable perceptual backbone similar to LPIPS, but introduces a geodesic distance-driven margin within a triplet contrastive formulation. Positive and negative DRR samples are generated using a differentiable renderer with controlled pose perturbations, explicitly linking perceptual similarity to rigid transformation discrepancies. This design encourages the network to learn geometry-consistent, deformation-free embeddings. Moreover, a dynamic pose-dependent margin further stabilizes training across large perturbation ranges, enhancing capture range while preserving convexity. Integrated within a differentiable 2D/3D optimization framework, the resulting metric yields smooth and robust loss landscapes, improving convergence reliability in both single-plane and dual-plane fluoroscopic scenarios.

The remainder of this paper is organized as follows. Section Methods describes the datasets, the pose-driven DRR sampling strategy, the proposed FluoroLPIPS architecture, and its integration into a differentiable 2D/3D registration framework. Section Experiments and results presents the experimental evaluation, including loss landscape analysis and registration performance under randomized pose perturbations. Finally, the Discussion and Conclusion section summarizes the main findings, discusses the limitations of the current study, and outlines directions for future work.

## Methods

### Datasets

**Fig. 1:**
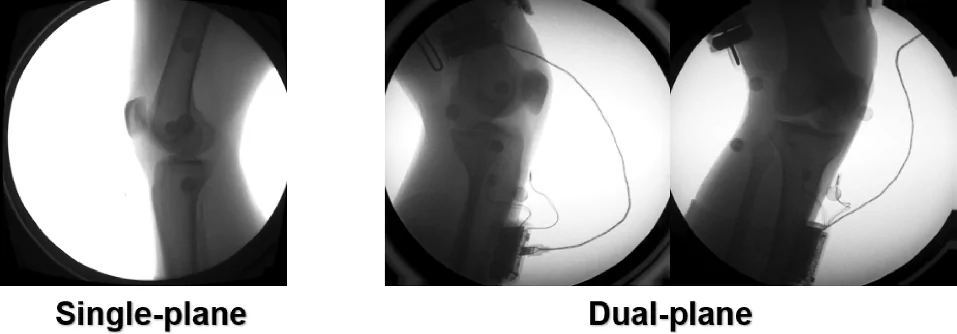
Fluoroscopic datasets collected using single- and dual-plane imaging systems

Two types of data were used in this study: (1) real fluoroscopic images acquired from in vivo motion trials using single-plane and dual-plane systems [32–34], and (2) synthetic digitally reconstructed radiographs (DRRs) generated using an in-house differentiable renderer.

Both single-plane and dual-plane real fluoroscopic datasets were employed for evaluation (Fig. 1). The single-plane fluoroscopy dataset was collected over the past ten years and includes data from 22 subjects performing four types of motion trajectories: deep knee bending, standing, squat, and knee flexion [33]. These data were acquired using a single-plane tracking fluoroscopic system [32] and manually annotated by trained operators to provide ground-truth poses. Due to its sufficient scale and annotation quality, this dataset was used for training and validation of the proposed deep similarity metric.

The dual-plane fluoroscopy dataset was collected more recently using a state-of-the-art dual-plane tracking system [34] with healthy subjects for Zimmer study (Zimmer Biomet). Compared to the single-plane system, the dual-plane setup employs a larger focal length (approximately 1870 mm versus 960 mm), resulting in increased noise levels and stronger artifact occlusions due to the more complex experimental configuration. Only a limited number of dual-plane images were manually annotated with high confidence; therefore, this dataset was used exclusively for zero-shot testing to evaluate the generalization capability of the proposed method across different imaging devices. Images from both datasets contains markers from our Vicon Nexus system for motion analysis. Although the geometric relationship between the two fluoroscopic planes is available, we intentionally used only the frontal plane during evaluation to isolate the contribution of the proposed similarity metric and to enable a fair single-view comparison against all baseline similarity measures. Extending the framework to coupled 3D/2×2D optimization is an important direction for future work.

Synthetic DRRs were generated from pre-segmented subject-specific bone models (femur or tibia only), rather than from the full CT volume including soft tissues. The renderer is a self-designed differentiable renderer [35, 36] based on Pytorch3D. The rendered images were produced at a resolution of 512×512 pixels to balance registration accuracy and computational efficiency. Realistic imaging noise, including Gaussian and salt-and-pepper noise, was added in training to improve robustness. These DRRs served as positive and negative samples during contrastive training, depending on whether perturbed or ground-truth poses were applied.

The reference poses for both the single-plane and dual-plane fluoroscopy datasets were established by manual model–image alignment using in-house software operated by trained annotators. Because no precise anatomical landmarks are directly available in the fluoroscopic images for exact pose determination, these manually aligned poses were used as reference poses throughout this study rather than absolute ground truth. To reduce annotation uncertainty, each registration was reviewed by multiple experienced raters. Training was conducted exclusively on the single-plane fluoroscopy dataset. The data were randomly split into training, validation, and testing sets in a ratio of 6:2:2. All anatomy models and images used for testing, including all dual-plane data and the held-out single-plane test set, were strictly excluded from training.

### Pose-aware perceptual similarity

#### Pose-driven DRR sampling

**Fig. 2:**
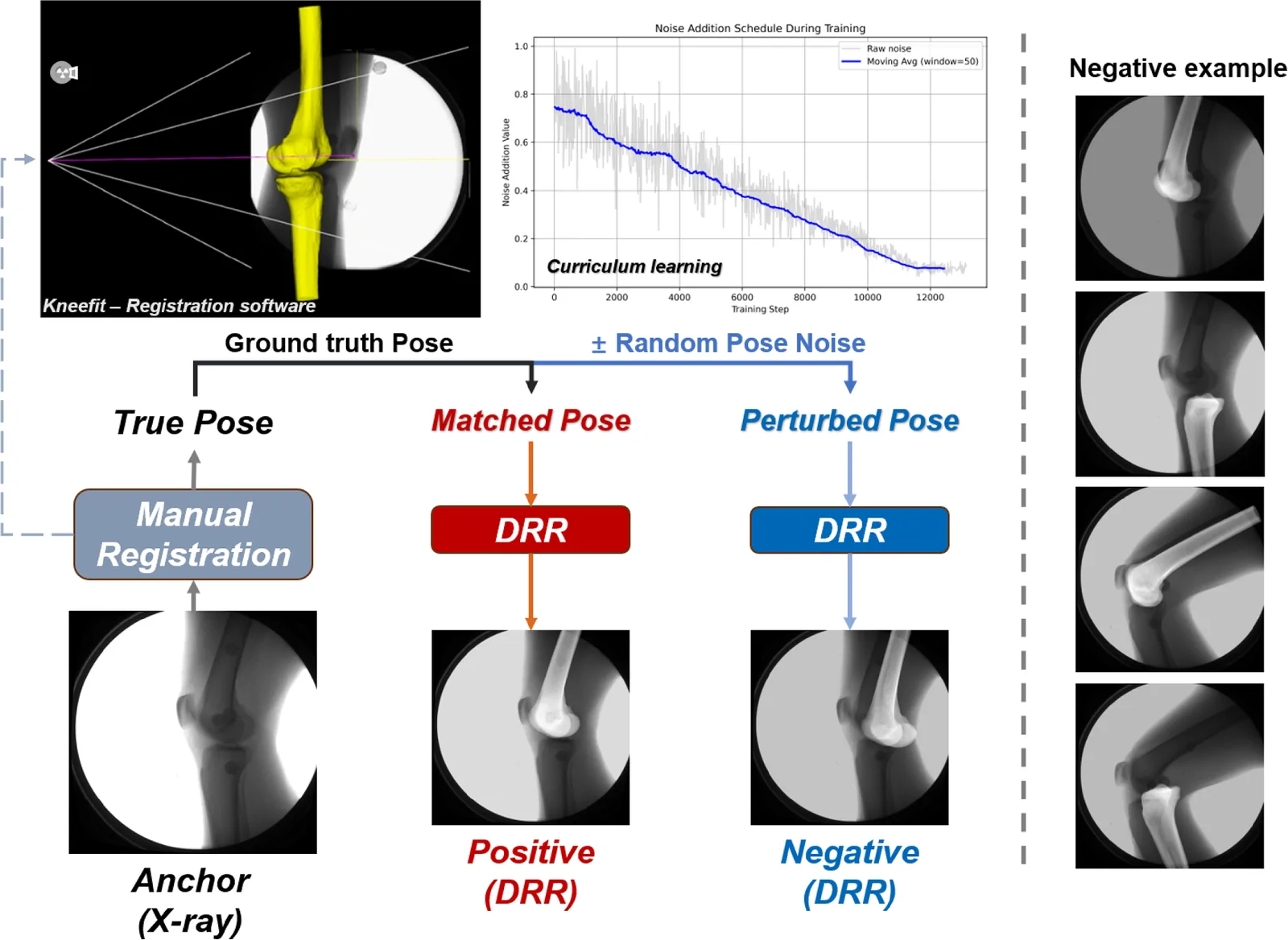
Pose-driven contrastive sampling using fluoroscopic images as anchors and DRRs rendered at true/perturbed poses as positive/negative samples, with perturbation magnitudes gradually reduced following a curriculum learning strategy

For each fluoroscopic frame, positive and negative samples are generated using a differentiable DRR renderer driven by controlled rigid 3D pose perturbations (translations and rotations) of the reference pose (Fig. 2). Real fluoroscopic images are used exclusively as anchors to avoid domain shift. Positive DRR samples are rendered at the unperturbed reference pose, preserving anatomical alignment and producing radiographs that are visually close to the anchor. Negative samples are rendered by applying pose perturbations that introduce geometric misalignment.

For each fluoroscopic frame, positive and negative samples were generated using a differentiable DRR renderer driven by controlled rigid 3D pose perturbations of the manually established reference pose described in Section Datasets. Real fluoroscopic images were used exclusively as anchors to avoid domain shift. Positive DRR samples were rendered at the unperturbed reference pose, preserving anatomical alignment and producing radiographs that were visually close to the anchor. Negative samples were rendered by applying pose perturbations that introduced geometric misalignment.

**Fig. 3:**
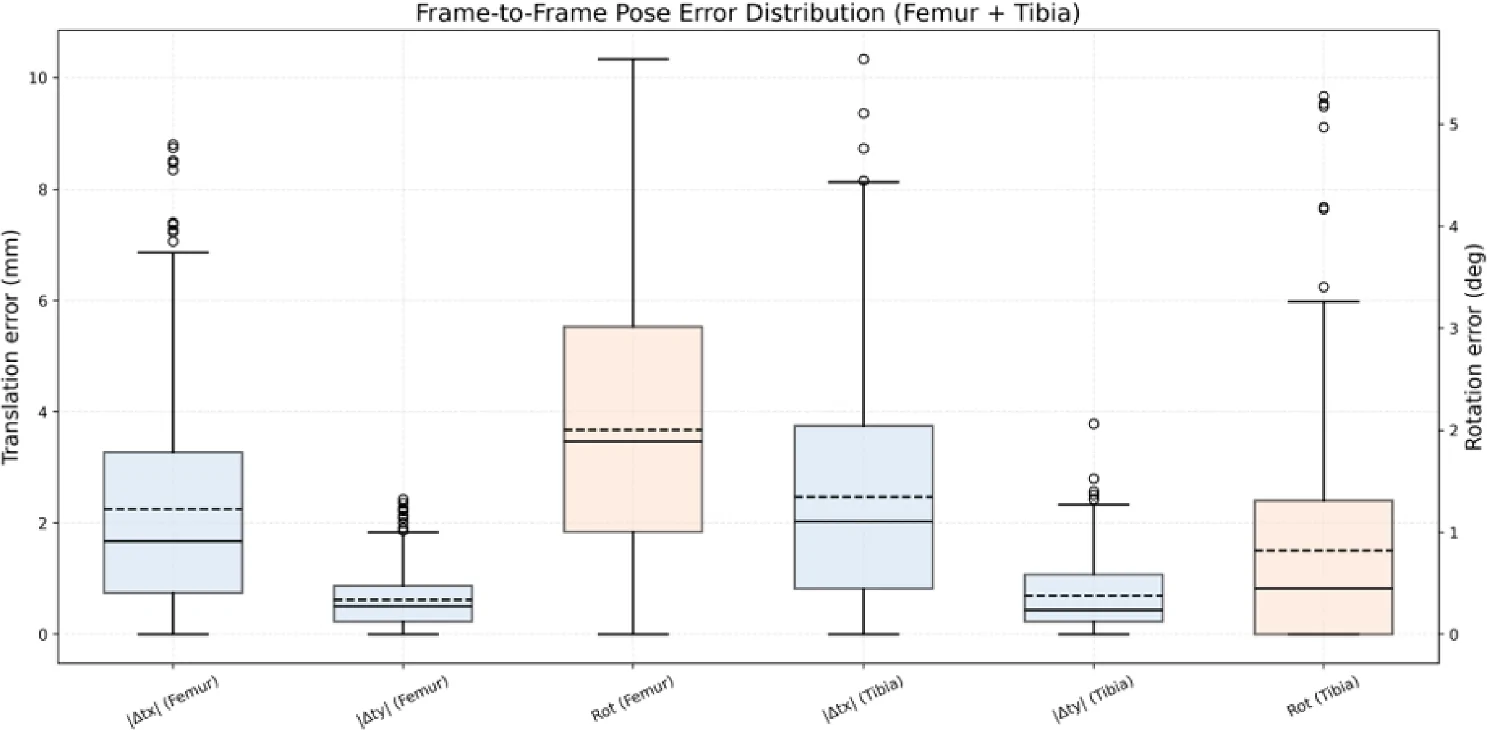
Statistics of inter-frame pose differences in fluoroscopic motion sequences

Both translational and rotational perturbations were applied with respect to the centroid of the target bone model. Translational perturbations were bounded within ±10 mm. Rotational perturbations were sampled in axis–angle form, where a random unit axis was drawn uniformly and the rotation magnitude was limited to a maximum geodesic distance of $10^\circ$, after which the perturbation was converted to a rotation matrix for rendering. Figure 3 reports the empirical distribution of inter-frame pose differences between consecutive manually annotated fluoroscopic frames. The perturbation bounds of ±10 mm and $10^\circ$ were chosen based on these observed frame-to-frame pose variations, with the aim of reflecting a realistic operating range for sequential registration.

We note that translational and rotational perturbations were not explicitly normalized to produce equivalent projected image displacement in the fluoroscopic image plane. Instead, the perturbation range was chosen in a data-driven manner based on realistic motion statistics from the target application. A decreasing random perturbation drift was further applied during training as a curriculum strategy, allowing the network to learn coarse pose discrepancies before focusing on finer alignment differences.

#### Model structure

**Fig. 4:**
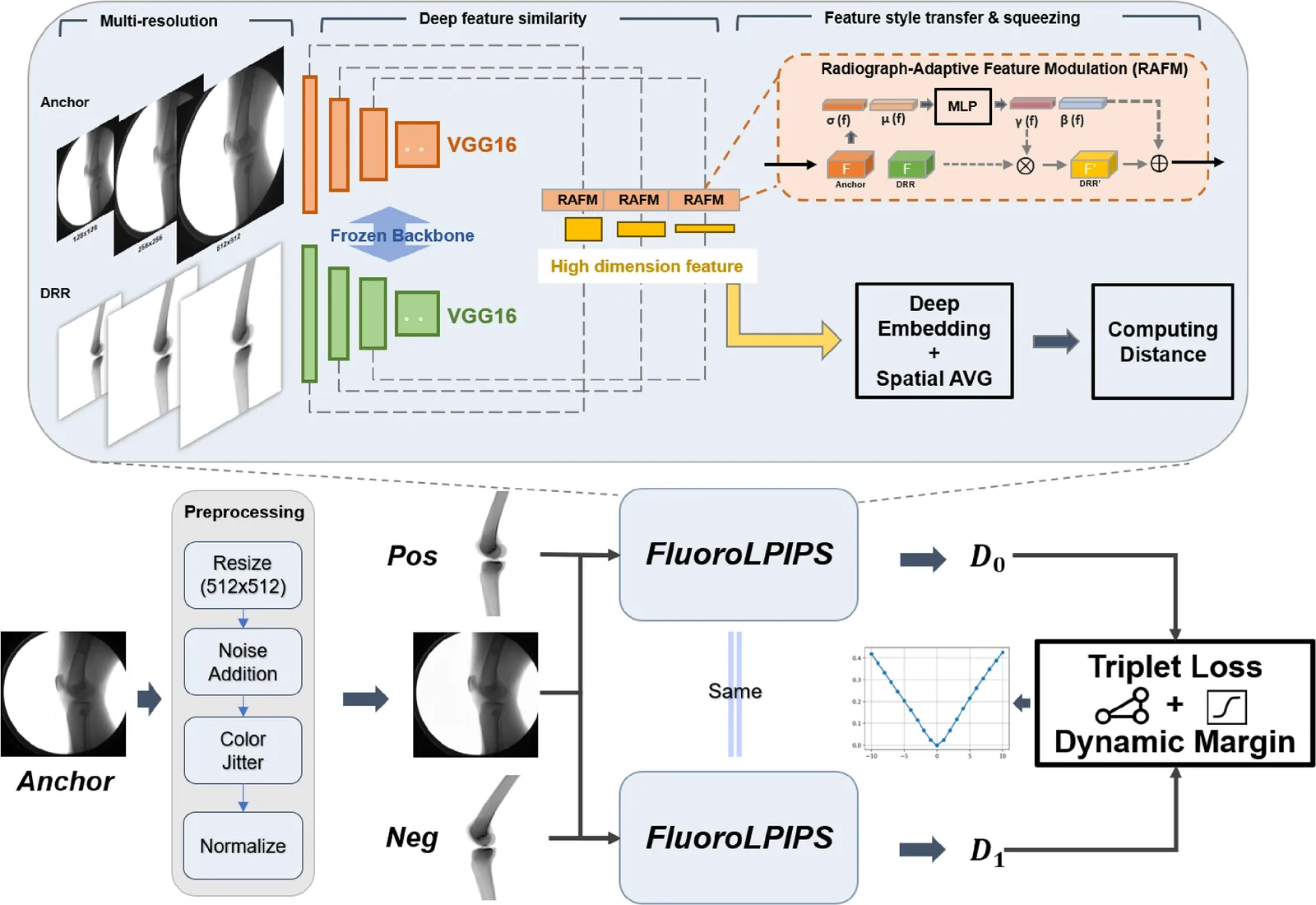
Architecture of the proposed FluoroLPIPS network and its pose-aware contrastive learning framework for perceptual similarity learning. The figure illustrates the overall learning framework schematically; in practice, femur and tibia were processed separately as independent rigid registration targets

To bridge the appearance gap between fluoroscopic images and DRRs while retaining sensitivity to rigid pose discrepancy, we built FluoroLPIPS on a VGG16 backbone and computed perceptual similarity from selected multi-scale feature layers (Fig. 4). In the current implementation, similarity was evaluated using all five highest resolution feature stages, which were found to provide the best trade-off between structural sensitivity and computational efficiency for iterative registration. A dedicated radiograph-adaptive feature module (RAFM) was attached to each selected feature stage before distance computation.

Although the domain gap between fluoroscopic images and DRRs is reduced in deeper feature representations, residual modality differences remain due to distinct radiographic contrast, attenuation behavior, and acquisition artifacts. The RAFM was introduced to adapt the extracted features to this fluoroscopy-to-DRR comparison setting before computing perceptual similarity. In addition, both the anchor fluoroscopic image and the rendered DRR were processed at multiple input scales to improve robustness to structural differences across resolution.

The training of FluoroLPIPS uses a pose-aware contrastive learning to adapt its feature space to pose-related differences. The training of our metric takes around 2 days to converge on an NVIDIA RTX4090 GPU. Only the *lin* configuration in training is employed to ensure a stable performance [23].

To better reflect true geometric separation, we introduce a triplet loss with dynamic margin that adapts to the SE(3) (i.e., 3D translation and rotation groups) discrepancy between the anchor pose and the sampled DRR pose (Eq. (1)). Let *a*, *p*, and *n* denote the anchor image, positive DRR, and negative DRR, respectively, and let $d(\cdot ,\cdot )$ denote the perceptual distance in the learned feature space.

1 $$\begin{aligned} \mathcal {L}_{\text {triplet}} = \max \left( d(a,p) - d(a,n) + m_{\text {dyn}},\, 0 \right) , \end{aligned}$$

where the margin is dynamically adjusted according to the pose discrepancy,

2 $$\begin{aligned} m_{\text {dyn}} = \alpha \cdot d_{\text {geo}}, \end{aligned}$$

with $d_{\text {geo}}$ denoting the combined SE(3) geodesic distance and α a scaling coefficient.

Let $f_l(\cdot )$ denote the feature map extracted from the lth selected backbone layer after the corresponding RAFM, and let $\hat{f}_l(\cdot )$ denote its channel-wise normalized representation. The perceptual distance between two images x and y is defined as

3 $$\begin{aligned} d(x,y)=\sum _{l \in \mathcal {L}} w_l \cdot \frac{1}{H_lW_l}\sum _{h,w}\left\| \hat{f}_l(x)_{hw}-\hat{f}_l(y)_{hw}\right\| _2^2, \end{aligned}$$

where $\mathcal {L}$ is the set of selected feature layers, $H_l$ and $W_l$ are the spatial dimensions of layer l, and $w_l$ denotes the learned linear weight for that layer.

This formulation follows the LPIPS distance design in spirit, but the network is retrained for fluoroscopy-to-DRR comparison using the proposed pose-aware triplet supervision, and is therefore task-specific rather than a direct use of the original LPIPS model.

In this study, the femur and tibia were treated as independent rigid bodies and processed separately throughout both training and registration. Accordingly, the rendered DRRs corresponded to the target bone being aligned, rather than to a coupled femur–tibia joint model.

### Differentiable registration framework

**Fig. 5:**
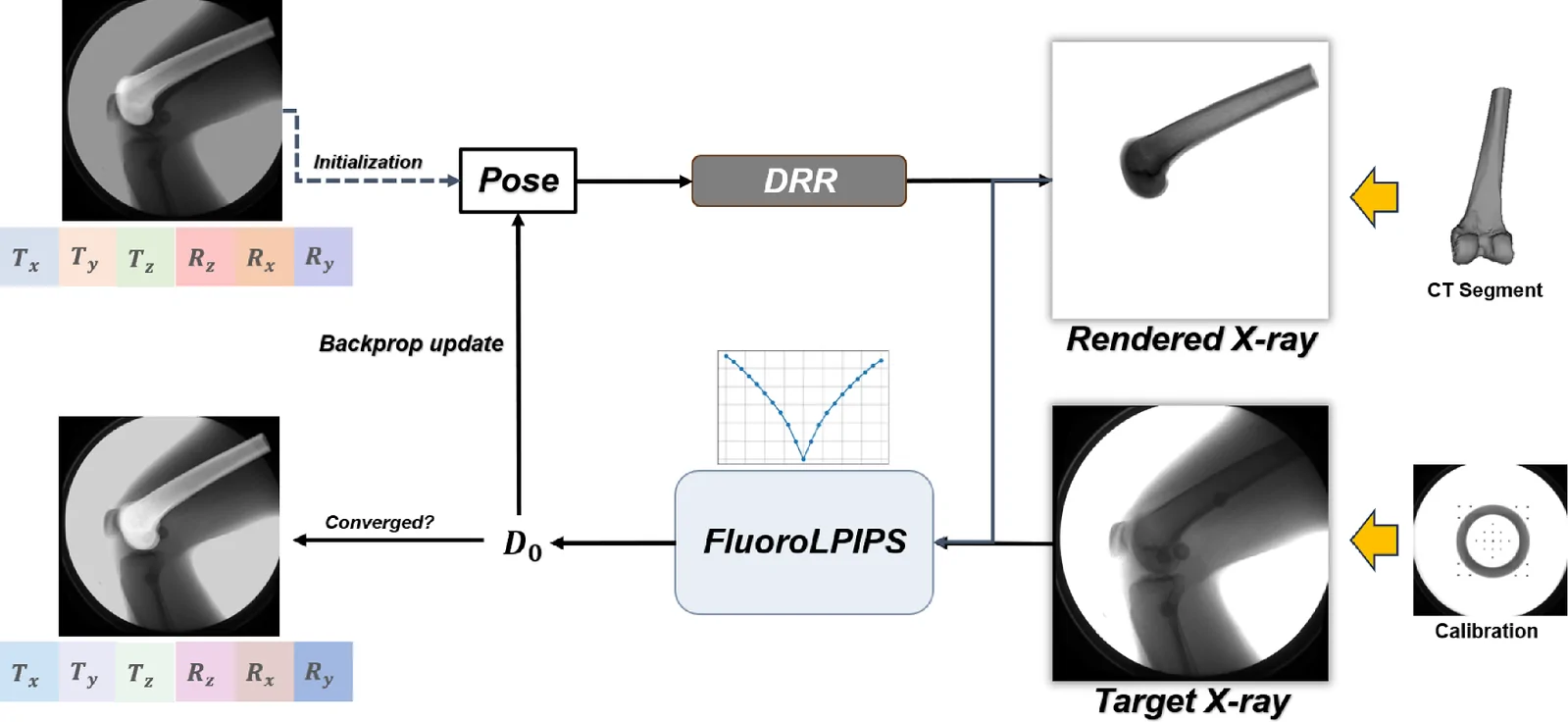
Iterative 2D/3D registration using FluoroLPIPS and differentiable renderer

Once training was completed, the network parameters were fixed. The learned similarity metric was then embedded into a fully differentiable 2D/3D registration framework using a PyTorch3D-based renderer. Given an initial pose estimate, the renderer generates DRRs that are iteratively aligned to the real fluoroscopic image by minimizing the learned perceptual distance (Fig. 5).

Given a target fluoroscopic image $I_{\textrm{fluoro}}$ and pose parameter $\theta \in SE(3)$, the registration objective is defined as

4 $$\begin{aligned} \mathcal {L}(\theta )=d\big (I_{\textrm{fluoro}}, R(M,\theta )\big ), \end{aligned}$$

where R(M,θ) denotes the DRR rendered from the target bone model M at pose θ and $d(\cdot ,\cdot )$ is the learned perceptual distance. Because both the renderer R and the similarity metric d are differentiable, gradients $\nabla _\theta \mathcal {L}(\theta )$ can be obtained by automatic differentiation and used for iterative optimization. These empirical properties are beneficial for gradient-based optimization, as they provide more stable descent behavior during iterative registration.

The optimization pipeline operates in SE(3) and updates translation and rotation jointly using AdamW. For the dual-plane dataset, only the frontal image was used in the registration pipeline to enable a fair single-view comparison. Notably, the similarity metric was trained only on single-plane data.

## Experiments and results

### Loss landscape analysis

To investigate whether the proposed pose-aware similarity yields a smooth and optimizable objective function, we first analyze its loss landscape [37, 38] under controlled perturbations. For each fluoroscopic frame, we generate a set of DRRs by systematically sweeping one pose parameter while keeping all others fixed. Representative 1D and 2D slices of the full 6-DoF registration landscape were evaluated, including in-plane translation and rotation dimensions that are particularly relevant for visualizing local optimization behavior around the reference pose. Both translational (±10 mm) and rotational (±10$^{\circ }$) dimensions were evaluated. For each sampled pose, the similarity value between the DRR and the real X-ray anchor is computed using GCC (gradient correlation coefficient), LNCC (local normalized cross-correlation), standard LPIPS, and our proposed FluoroLPIPS.

**Fig. 6:**
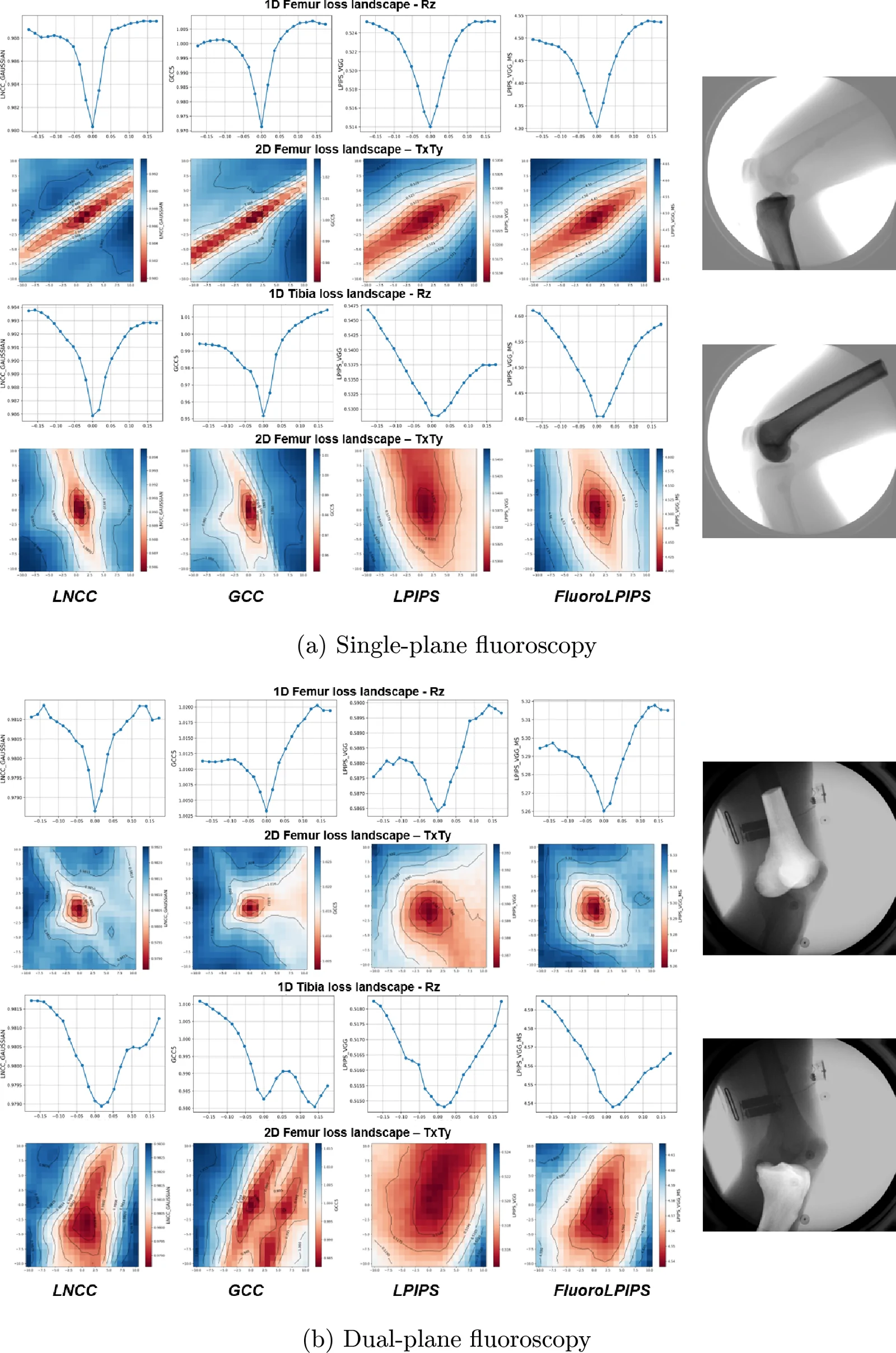
Representative 1D and 2D slices of the registration loss landscape for different similarity metrics. The plots show similarity variation under controlled perturbations of selected pose parameters around the manually established reference pose, while all remaining pose parameters are kept fixed. Translations are reported in millimeters and rotations in degrees

Across all subjects and anatomical structures, FluoroLPIPS consistently produced a smoother and more monotonic loss landscape within the evaluated perturbation range, with a clearer minimum around the reference pose and fewer obvious spurious local minima than the baseline similarity measures. (Fig. 6). In contrast, traditional metrics showed smooth gradients (Fig. 6a—GCC, Fig. 6b—LNCC), strong local minimum (Fig. 6b—GCC), narrow capture range smooth gradients (Fig. 6a—LNCC), and obscurity (Fig. 6—LPIPS). Standard LPIPS exhibited partial smoothness but lacked sensitivity to small geometric changes. FluoroLPIPS presents a smoother 2D loss landscape with a clearer minimum around the reference pose and fewer obvious spurious local minima within the evaluated perturbation range, also in low-contrast images (dual-plane). The resulting empirical loss landscape improves gradient-based optimization stability and enlarges the range of successful convergence around the reference pose.

**Fig. 7:**
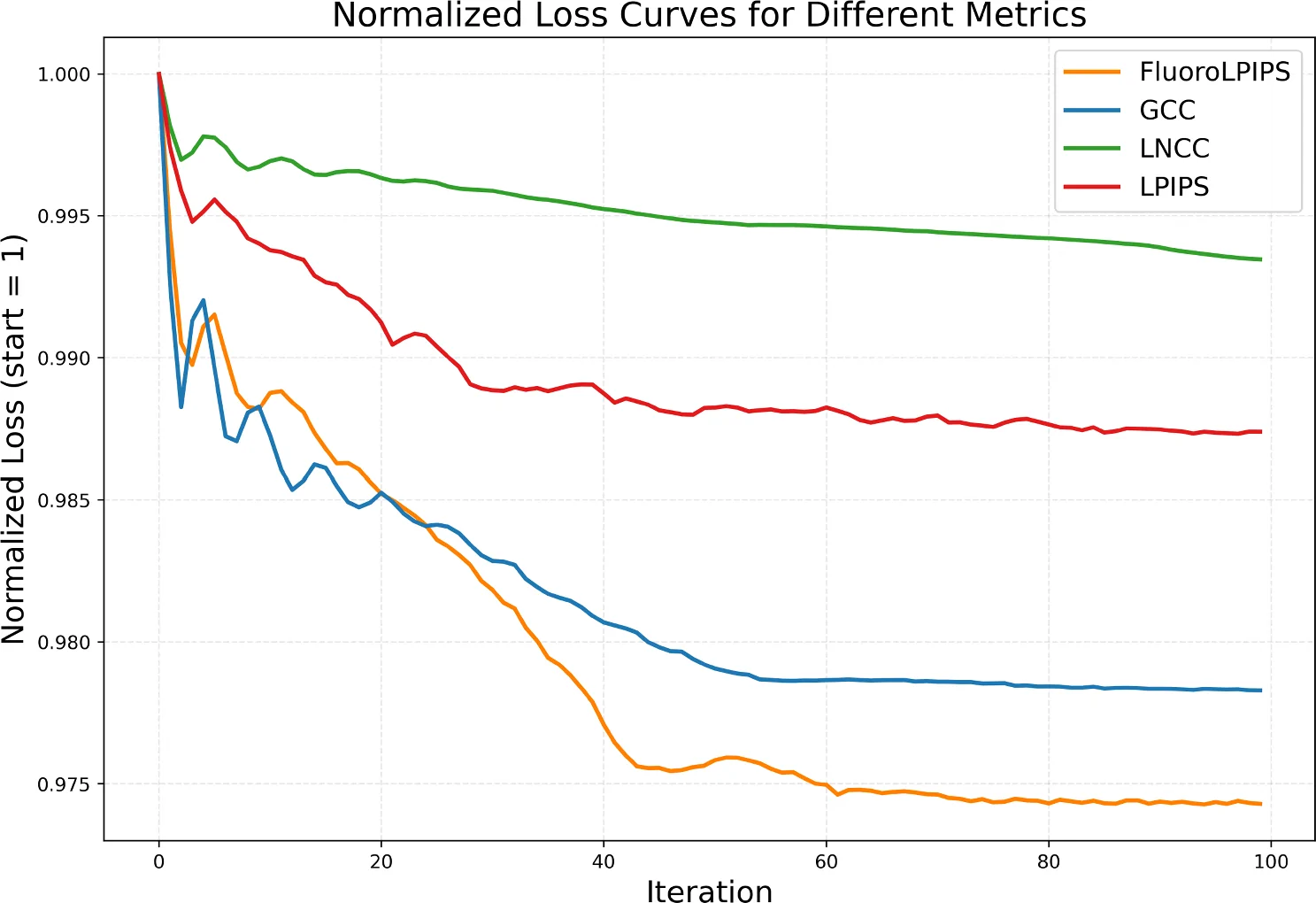
Optimization loss curves for iterative 2D/3D registration, starting from the same initial pose perturbation for 100 iterations with learning rate 0.2. The plotted loss was normalized by the initial loss value of each trial

A further analysis of the loss evolution starting from the same initial pose perturbation for different similarity measures (Fig. 7) shows that our metric presents largest pose sensitivity in depth, avoiding irregular oscillation and stagnation in optimization. Compared with conventional handcrafted similarity measures, FluoroLPIPS yields more stable and informative descent behavior during optimization, which is consistent with the smoother empirical loss landscape observed in the evaluated perturbation range.

### Registration performance

To quantify the practical impact of the proposed similarity metric on optimization-based 2D/3D registration, we conducted a large-scale random disturbance evaluation. For each fluoroscopic frame, one hundred randomized perturbations were generated by sampling in-plane translations up to ±10 mm and rotational offsets up to ±10$^{\circ }$, creating a diverse set of initialization conditions representative of real fluoroscopy-based motion capture scenarios (Fig. 3). Each perturbed pose was subsequently refined using the differentiable registration pipeline driven by GCC, LNCC, standard LPIPS, or the proposed FluoroLPIPS.

**Table 1:** Registration performance under random pose perturbations for femur and tibia on single-plane and dual-plane fluoroscopic data. Translation errors are reported per axis. Success rate (SR) is defined as final translation error <1 mm and rotation error $<1^\circ$

View	Anatomy	Similarity	$T_x$ (mm)	$T_y$ (mm)	$T_z$ (mm)	Rot ($^{\circ }$)	SR (%)
Single	Femur	GCC	2.21±1.34	2.05±1.26	2.78±1.92	2.02±2.41	84.8
		LNCC	2.84±1.02	1.73±0.98	2.51±1.73	2.36±2.12	78.1
		LPIPS	2.52±0.91	2.38±0.87	2.34±1.61	3.89±1.96	72.6
		FluoroLPIPS	**0.82±0.51**	**0.79±0.49**	**2.21±1.84**	**0.94±0.63**	**98**.**6**
	Tibia	GCC	2.35±1.41	2.18±1.33	2.11±2.04	2.28±2.53	85.2
		LNCC	1.96±1.12	1.84±1.06	2.63±1.81	2.47±2.21	75.4
		LPIPS	2.67±0.98	2.53±0.94	2.42±1.69	4.01±2.03	71.9
		FluoroLPIPS	**0.91±0.58**	**0.88±0.55**	**2.34±1.91**	**0.98±0.67**	**98**.**1**
Dual	Femur	GCC	2.98±1.62	2.84±1.58	3.42±2.11	5.08±2.91	69.5
		LNCC	2.56±1.38	2.41±1.34	3.16±1.96	5.32±2.48	75.3
		LPIPS	3.21±1.21	2.04±1.18	2.89±1.74	5.61±2.17	65.2
		FluoroLPIPS	**1.64±0.97**	**1.52±0.93**	**2.58±1.83**	**2.87±1.54**	**88**.**4**
	Tibia	GCC	2.14±1.71	2.97±1.66	3.61±2.24	5.41±3.02	70.8
		LNCC	2.71±1.49	2.56±1.45	3.28±2.01	4.64±2.61	72.9
		LPIPS	3.36±1.28	3.19±1.25	3.01±1.82	5.89±2.29	60.6
		FluoroLPIPS	**1.78±1.04**	**1.66±0.99**	**2.73±1.92**	**3.12±1.68**	**86**.**9**

For each trial, the final translation and rotation errors were computed with respect to the corresponding manually established reference pose. Translation errors were reported separately along the three translation axes, and rotational error was quantified using geodesic rotation distance. A registration trial was considered successful if the final translation error was below 1 mm and the final rotation error was below $1^\circ$. These submillimeter and sub-degree thresholds were chosen because such accuracy is typically required for fluoroscopy-based joint kinematic analysis to remain clinically and biomechanically meaningful.

Quantitative results in Table 1 demonstrate that FluoroLPIPS consistently outperforms all baseline similarity metrics across both anatomical structures and imaging configurations. On single-plane fluoroscopic data, FluoroLPIPS achieves submillimeter accuracy in the in-plane directions ($T_x$, $T_y$) together with sub-degree rotational error, resulting in success rates exceeding 98% for both femur and tibia under the stricter success criterion of 1 mm / $1^\circ$. In contrast, traditional metrics and standard LPIPS exhibit substantially larger errors and reduced convergence reliability under the same initialization conditions.

As expected, overall performance degrades on dual-plane data (zero-shot evaluation) due to differences in device, imaging modality, and increased noise. Nevertheless, FluoroLPIPS maintains a clear better performance than competing metrics, achieving significantly higher success rates and lower pose errors. These results indicate that the proposed similarity metric presents greater robustness to initialization uncertainty and noises.

**Fig. 8:**
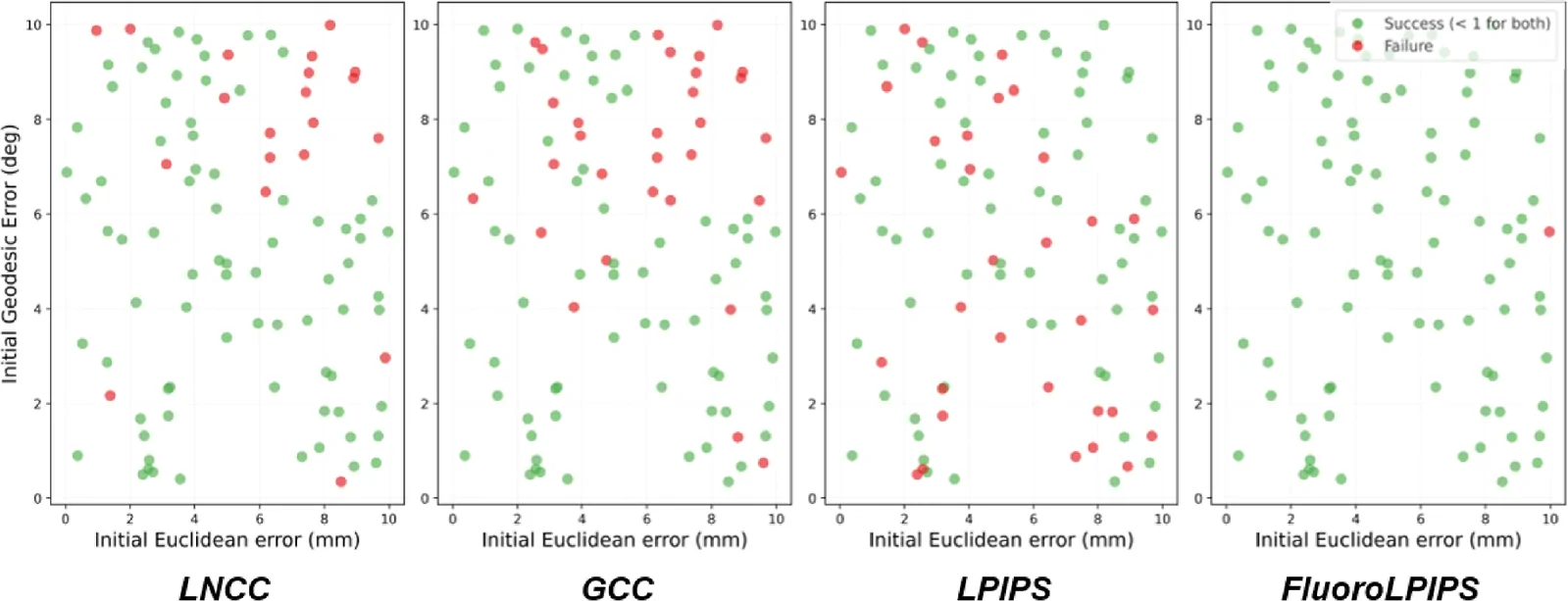
Empirical convergence behavior of different similarity metrics under randomized pose perturbations. Each point represents one registration trial, with successful convergence shown in green and failed convergence shown in red. The *x*-axis indicates the initial Euclidean translation magnitude of the perturbation, and the *y*-axis indicates the initial geodesic rotational error, both measured relative to the manually established reference pose

The improvement in convergence reliability is further illustrated in Fig. 8, which visualizes the empirical distribution of successful and failed registration trials under randomized initialization. Each point corresponds to an individual perturbation trial and is color-coded by success or failure. We emphasize that this figure is intended as an empirical illustration of convergence behavior rather than a formal characterization of the full capture range. Compared with the baseline metrics, FluoroLPIPS shows fewer obvious failure clusters under comparable perturbation magnitudes, suggesting a reduced tendency toward undesirable local minima in the evaluated perturbation range.

**Fig. 9:**
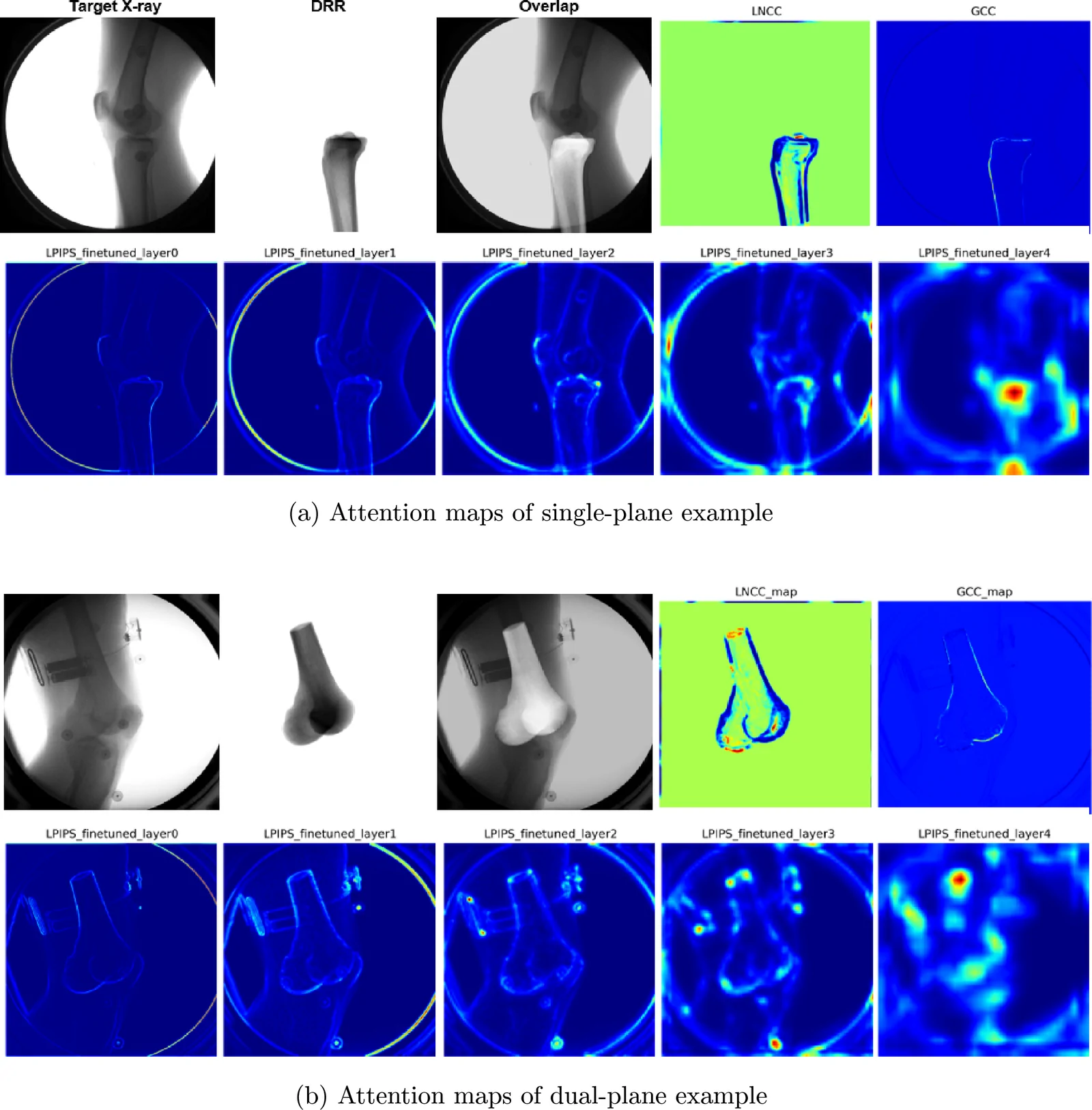
Heatmap visualizations of correlation responses for LNCC and GCC, together with multilayer feature activations of FluoroLPIPS

Visualizing the attention of similarity (Fig. 9) showed that metrics such as GCC and LNCC rely on low-level intensity correlation, resulting in diffuse or ambiguous response maps that are sensitive to noise and occlusion. In contrast, FluoroLPIPS produces structure-informed activation patterns across multiple feature layers, focusing more strongly on anatomically meaningful regions and capturing high-dimensional correspondences between DRRs and fluoroscopic images. We note that no explicit masking was applied outside the fluoroscopic field of view in the current implementation, which may contribute to the visible circular boundary in the response maps.

## Discussion–conclusion

This study examined the effect of incorporating pose awareness into a learned perceptual similarity metric for iterative fluoroscopy-to-CT 2D/3D registration. The results show that embedding pose supervision into the similarity function leads to improved loss landscape smoothness, convergence reliability, and effective capture range compared with conventional metrics and standard LPIPS.

Compared with traditional similarity measures, FluoroLPIPS consistently exhibits smoother and near-convex loss landscapes. Conventional metrics such as GCC and LNCC rely on fixed intensity-based or feature-based operations (e.g., gradient filters), which are sensitive to noise, occlusion, and contrast variations that are commonly present in fluoroscopic images. These effects often introduce non-convex loss surfaces and local minima, as also reported in previous optimization-based registration studies [15, 19, 22]. In contrast, the deep network can implicitly generalize such low-level operations while suppressing image variations that are not informative for geometric alignment, including Gaussian noise and occlusions caused by reflective motion markers (Vicon Nexus). Prior work has attempted to mitigate these issues by modifying traditional metrics through localization, patch-based strategies, weighted similarity formulations, or region-of-interest masking [19], but such approaches remain limited by hand-designed assumptions and fixed representations.

FluoroLPIPS substantially enlarges the effective capture range under random pose perturbations in optimization. While conventional metrics and standard LPIPS frequently fail to converge when initialized far from the true pose, FluoroLPIPS consistently converges across a broader range of initial conditions. This improvement reflects the ability of the learned metric to provide meaningful descent directions at large pose errors while maintaining sensitivity near the optimum. The geodesic-driven dynamic margin further enforces appropriate separation between aligned and misaligned poses in the similarity space. Such enlarged capture range is particularly important for constructing fully automated registration pipelines, where initialization is often provided by learning-based pose predictors [6, 7].

A further observation is the strong generalization capability of FluoroLPIPS across imaging configurations. Although trained exclusively on single-plane fluoroscopic data, the metric generalizes effectively to dual-plane acquisitions without additional fine-tuning. In contrast, many learning-based registration approaches are tightly coupled to specific imaging geometries or acquisition protocols [26, 31], limiting its application to many registration tasks though being invariant to modalities.

Several limitations should be acknowledged. First, the performance of the proposed metric depends on the accuracy of CT-derived anatomical models and imaging calibration. Moreover, the current method focuses on rigid single-bone registration, and extending the approach to multi-bone or implant–bone systems remains future work. Moreover, we did not explicitly evaluate additional metric enhancement strategies, such as multi-scale processing, patch-based similarity, or region-of-interest masking, which could potentially further improve robustness.

We also acknowledge that such manually established reference poses may still contain residual error, which is a common limitation in fluoroscopy-based 2D/3D registration studies. Although each registration was reviewed by multiple experienced raters to reduce annotation uncertainty, intra- and inter-expert variability was not explicitly quantified in the present study. A systematic assessment of rater variability would provide an additional reference for interpreting the accuracy of automated registration methods and will be considered in future work.

Future work may use the proposed similarity metric to learning-based reconstruction and registration frameworks, where similarity plays a central role in supervision and optimization [39–41]. Further improvements could be improving architecture or utilizing multi-view and temporal fluoroscopic sequences. In addition, the current implementation does not explicitly mask or down-weight the region outside the fluoroscopic field of view. Although all compared metrics were evaluated under the same input conditions, suppressing this non-anatomical region may further improve the robustness and interpretability of the learned similarity in future work.

Overall, this study demonstrates that learning a pose-aware deep perceptual similarity offers an effective alternative to traditional similarity measures and generic perceptual losses for optimization-based 2D/3D registration. By aligning perceptual similarity with rigid transformation discrepancies through contrastive pose supervision, FluoroLPIPS, as a plug-and-play metric, improves optimization behavior while preserving the flexibility of iterative registration frameworks.
